# Modulation of Protein Synthesis by eIF2α Phosphorylation Protects Cell from Heat Stress-Mediated Apoptosis

**DOI:** 10.3390/cells7120254

**Published:** 2018-12-07

**Authors:** Soyoung Park, Yohan Lim, Duckgue Lee, Rosalie Elvira, Ji-Min Lee, Man Ryul Lee, Jaeseok Han

**Affiliations:** Soonchunhyang Institute of Medi-bio Science (SIMS), Soonchunhyang University, Cheonan-si, Chungcheongnam-do 31151, Korea; ivydawn92@gmail.com (S.P.); yohan.lim@rwth-aachen.de (Y.L.); tomyoo27@gmail.com (D.L.); maria.rosalie.elvira@gmail.com (R.E.); jmajw@hanmail.net (J.-M.L.)

**Keywords:** heat stress, ER stress, translation, eIF2α phosphorylation, unfolded protein response (UPR)

## Abstract

Global warming poses a considerable threat to human health, necessitating a proper understanding of mechanisms underlying cell death in the pathogenesis of heat-related diseases. Although mechanisms governing cytoplasmic response to heat are well understood, processes regulating cellular response to disruption of proteostasis in the endoplasmic reticulum (ER) due to heat stress remain unclear. The current study reveals that hyperthermic conditions may lead to a disturbance of ER homeostasis, also known as ER stress. Subsequent activation of the unfolded protein response (UPR) resulted in concomitant induction of cell death. Among the three UPR signaling pathways, the eIF2α phosphorylation pathway, and not the IRE1α/ATF6α pathways, is likely the main contributor to cell death under heat stress. Considering the role of eIF2α in translational control, we investigated the protective effect of translation rate on heat stress-mediated cell death. When protein synthesis was attenuated using cycloheximide or homoharringtonine, cell death due to heat stress was significantly reduced. In summation, we propose that transient modulation of protein synthesis by eIF2α phosphorylation has a pivotal role in protecting cells from heat stress-induced apoptosis. Therefore, pharmacological agents that promote eIF2α phosphorylation or reduce ER stress may contribute to the development of promising therapeutic approaches against heat-related diseases.

## 1. Introduction

Global surface temperatures are on the rise and heat waves are projected to increase in frequency, duration, and intensity [1]. Global warming clearly poses a threat of considerable magnitude to human health [2]. Heatstroke, a typical heat-related illness, is characterized by increased body temperature greater than 40 °C and occurs because of excessive environmental heat and/or excessive metabolically produced heat. Although it is accompanied by death of organs, including the central nervous system, the exact mechanism of cell death and tissue injury during heatstroke is poorly understood. 

Protein homeostasis (proteostasis) is the process that controls the biogenesis, folding, trafficking, and degradation of proteins within cells [3]. It is reported that excessive heat causes proteostasis disturbance in cells by inhibiting protein folding and denaturing existing proteins, resulting in the accumulation of misfolded and/or unfolded proteins [4]. Since proteostasis disturbance is detrimental, cells evolved defensive mechanisms that restore homeostasis in order to minimize damage.

In the cytosol, the heat shock response (HSR) is mediated by a single transcription factor, heat shock factor 1 (HSF1) [5]. Under normal conditions, HSF1 is sequestered in a complex containing the chaperone, Hsp90 [6,7]. Upon exposure to heat, increasing levels of unfolded proteins compete for Hsp90, causing HSF1 to be released from the Hsp90 complex. Free active HSF1, in turn, enhances the transcription of heat shock genes that encode cytoplasmic chaperones including HSPA1A (HSP70) and DNAJB1 (HSP40) [6].

In the endoplasmic reticulum (ER), the unfolded protein response (UPR) is mediated by three transmembrane proteins: PKR-like eukaryotic initiation factor 2α kinase (PERK), inositol-requiring enzyme 1α (IRE1α), and activating transcription factor 6α (ATF6α) [8]. In unstressed cells, these mediators are sequestered by a major ER chaperone, GRP78, also known as BiP [9,10,11]. When stressed, accumulated unfolded proteins compete for GRP78, resulting in the release of those aforementioned proteins and the subsequent induction of downstream signaling pathways. This classic mechanism is challenged by findings that show that the dissociation of IRE1α or PERK from GRP78 is not sufficient for UPR induction. In fact, direct interaction between unfolded or misfolded proteins and the UPR sensors might be required. Activated PERK phosphorylates the alpha subunit of the eukaryotic translation initiation factor 2 (eIF2α) at Ser51, leading to rapid and transient translational attenuation [12,13,14]. Paradoxically, translation of several mRNAs, including activating transcription factor 4 (ATF4), is preferentially enhanced [15,16]. IRE1α has dual cytosolic activities of a serine/threonine kinase and endoribonuclease [17]. The activated IRE1 α cleaves the mRNA encoding X-box binding protein 1 (XBP1) to produce a transcriptionally active form (XBP1s) [18,19]. ATF6α is a type II transmembrane protein that contains a cytosolic cAMP-responsive element-binding protein (CREB)/ATF basic leucine zipper (bZIP) domain. Once released from BiP, ATF6α traffics to the Golgi complex where it is cleaved to produce active forms of transcription factor that enter the nucleus to induce their target genes [20,21].

Accumulating evidence indicates that ER stress and subsequent induction of the UPR plays an important role in apoptotic cell death under several types of stress conditions [22]. Activated IRE1α interacts with the adaptor protein TRAF2 to activate JNK, leading to cell death [23]. It was also reported that IRE1α indiscriminately degrades ER-localized mRNA that can lead to cell death. Enhanced expression of ATF4 and CHOP coordinately enhances translation rate, which leads to cell death [24]. By contrast, proper induction of the UPR genes is crucial for protecting cells from stress, by restoring ER homeostasis. For example, inhibition of eIF2α phosphorylation or of IRE1α knock-out increases the susceptibility of cells to many stresses in pancreatic β-cells and hepatocytes [25,26,27,28].

In the present study, we investigated the role of ER stress and the UPR signaling pathways in heat-mediated cellular apoptosis with particular reference to elucidating the UPR branch that plays a pivotal role in protecting cells from heat stress.

## 2. Martials and Methods

### 2.1. Cell Culture

Mouse embryo fibroblasts (MEFs), including *Eif2*α*^A/A^*, *Eif2*α*^S/S^*, *Perk*^+/+^, *Perk*^−/−^, *Ire1*α^−/−^, *Ire1*α^+/+^, *Atf6*α^−/−^, and *Atf6*α^+/+^ were maintained in Dulbecco’s modified Eagle’s medium (DMEM, Invitrogen, Carlsbad, CA, USA) supplemented with 10% fetal bovine serum (FBS) and 1% of penicillin/streptomycin as described earlier [29]. MEFs were incubated at 37 °C in humidified 5% CO_2_ incubator for maintenance. To induce heat stress, MEFs were incubated at 42 °C in humidified 5% CO_2_ incubators for indicated times. Tunicamycin (Tm, Sigma, St. Louis, MO, USA, T7765), thapsigargin (Tg, Sigma, St. Louis, MO, USA, T9033), tauroursodeoxycholic acid (TUDCA, Calbiochem, St. Louis, MO, USA, 580549), cycloheximide (Sigma, St. Louis, MO, USA, C6255), homoharringtonine (Sigma, St. Louis, MO, USA, SML1091), guanabenz (GA, Sigma, St. Louis, MO, USA, G110) ISRIB (Sigma, St. Louis, MO, USA, SML0843), GSK2606414 (Tocris, Bristol, England, 5107), and 4μ8c (Cayman, Ann Arbor, MI, USA, 22110) were utilized as described in the text.

### 2.2. RNA Extraction and Real-Time PCR Analysis

Total RNA was extracted from cells using Trizol (Gene All, Ribo-Ex, Seoul, Korea) and cDNA was synthesized using an iScript cDNA synthesis kit (Bio-Rad, Hercules, CA, USA, BR170-8891). The relative amounts of mRNAs were calculated from the comparative threshold cycle (Ct) values relative to 18s rRNA using CFX96 Real-Time PCR detection system (Bio-Rad, Hercules, CA, USA, 184-5384) with SYBR green reagent (Enzynomics, Daejeon, Korea, RT500M) according to manufacturer’s instruction. Real-time primer sequences used in this study were as follows: Chop (5′-CTG CCT TTC ACC TTG GAG AC-3′; 5′-CGT TTC CTG GGG ATG AGA TA-3′), sXBP1 (5′-GAG TCC GCA GCA GGT G-3′; 5′-GTG TCA GAG TCC ATG GGA-3′), total XBP1 (5′-AAG AAC ACG CTT GGG AAT GG-3′; 5′-ACT CCC CTT GGC CTC CAC-3′), Grp78 (5′-GGT GCA GCA GGA CAT CAA GTT-3′; 5′-CCC ACC TCC AAT ATC AAC TTG A-3′), Hsp70 (5′-TCG AGG AGG TGG ATT AGA GG-3′; 5′-GCA GCT ATC AAG TGC AAA GAG-3′).

### 2.3. Western Blotting

Cell lysates were obtained in cell lysis buffer [50 mmol/L Tris, HCl (pH 7.4), 150 mmol/L NaCl, 1% (*v*/*v*) Triton X-100, 0.1% (*w*/*v*) SDS, 1% (*w*/*v*) sodium deoxycholate and protease/phosphatase inhibitors (Complete, Roche Diagnostics, Risch-Rotkreuz, Switzerland)], and total protein concentration in each sample was measured using a Bicinchoninic acid assay kit (Thermo Fisher Scientific, Waltham, MA, USA). Primary antibodies were as follows: p-eIF2α (Abcam, Cambridge, England, ab32157), eIF2α (Cell Signaling technology, Danvers, MA, USA, #9722), CHOP (Santacruz, Dallas, TX, USA, sc-575), ATF4 (Cell Signaling technology, Danvers, MA, USA, #11815), HSP70 (Enzo Life Sciences, Farmingdale, NY, USA, SPA822), cleaved Caspase-3 (Cell Signaling technology, Danvers, MA, USA, #9661), PARP (Cell signaling technology, Danvers, MA, USA, #9542), and α-tubulin (Sigma, St. Louis, MO, USA, T9026). Secondary antibodies were as follows: Peroxidase-conjugated affinipure goat anti-mouse IgG (Jackson laboratory, Bar harbor, ME, USA, 115-035-003) and anti-rabbit IgG (Jackson laboratory, Bar harbor, ME, USA, 111-035-003). Chemiluminescence detection using enhanced chemiluminescence (Thermo Fisher Scientific, Waltham, MA, USA) was performed. Membranes were exposed to imaging film and developed using an automatic film processor (Agfa, Mortsel, Belgium, CP1000).

### 2.4. Cell Viability Assays

Relative cell viabilities were measured using thiazolyl blue tetrazolium bromide (MTT) (Sigma, St. Louis, MO, USA, M2128). The final concentration of MTT solution is 0.5 ug/mL in complete media, followed by a 1 h incubation at 37 °C. DMSO was used to dissolve formazan after incubation. Photometric data was analyzed by a micro-plate reader (Promega, Madison, WI, USA). 

### 2.5. Immunofluorescence

Cells were cultured on sterile cover glass for 2 days, followed by treatment described in the text. Cells were fixed with MeOH at −20 °C for 5 min, followed by blocking with PBS-TX [PBS(Phosphate-buffered saline) with 5% Triton X-100] for 1 h at room temperature. After one day incubation at 4 °C with first antibodies (KDEL, Abcam, Cambridge, England, Ab12223), a secondary antibody (Alexa 488, Jackson laboratory, Bar harbor, ME, USA, 111-545-003) was used for identification. DAPI (Life technologies, Waltham, MA, USA, D1306) was used for nuclear staining. Observation was through L710 confocal microscope performed (Zeiss, Oberkochen, Germany).

### 2.6. Measurement of Protein Synthesis Rate

Following treatment, puromycin (10 μg/mL) was added for 10 min. Cell lysates were obtained in cell lysis buffer [50 mmol/L Tris, HCl (pH 7.4), 150 mmol/L NaCl, 1% (*v*/*v*) Triton X-100, 0.1% (*w*/*v*) SDS, 1% (*w*/*v*) sodium deoxycholate and protease/phosphatase inhibitors (Complete, Roche Diagnostics, Risch-Rotkreuz, Switzerland)], and the total protein concentration in each sample was measured using a Bicinchoninic acid assay kit (Thermo Fisher Scientific, Waltham, MA, USA). Incorporated puromycin was detected using anti-puromycin antibody (Millipore, Burlington, MA, USA, MABE343).

### 2.7. Measurement of Caspase 3/7 Activity

Cells (8 × 10^3^ cells/well) were incubated in a humidified 5% CO_2_ incubator for 12 h in 96-well white plate. Caspase-Glo 3/7 assay kit (Promega) was used to measure the caspase-3 and -7 activities according to the manufacturer’s instruction. Following incubation, 50 µL of Caspase-Glo 3/7 reagent was added and the plate was maintained at room temperature for 1 h prior to luminescence detection. Luminescence was calculated using a micro-plate reader (Promega).

### 2.8. Statistical Analysis

Data are represented as mean ± SEM. Statistical significance of differences between groups was evaluated using the Student *t* test. *p* values less than 0.05 were considered statistically significant.

## 3. Results

### 3.1. Heat Exposure Induces ER Stress-Mediated Apoptosis

**Figure 1:**
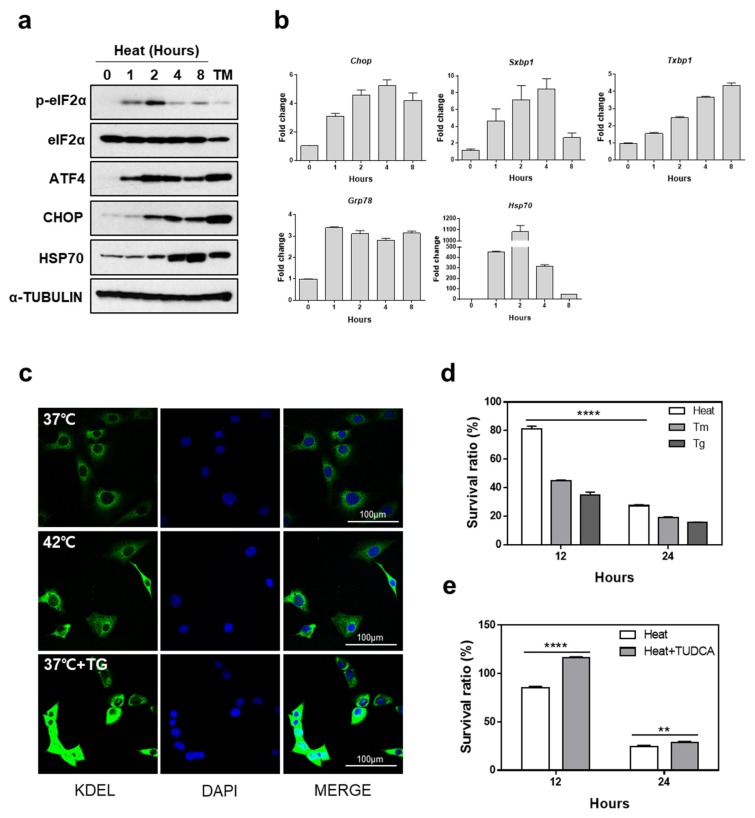
High temperature induces endoplasmic reticulum (ER) stress-mediated cell death. (**a**) Protein expression under condition of hyperthermia was measured. Cell lysates were collected at indicated times following incubation at 42 °C or tunicamycin (Tm) (1 μg/mL) treatment for western blot analysis. (**b**) Effect of heat stress on the unfolded protein response (UPR) gene expression. Quantitative RT-PCR was performed using total RNA extracted from mouse embryonic fibroblasts (MEFs) which were incubated at 42 °C for indicated time periods. Data are presented as means ± SEM (n = 3 independent experiments). (**c**) KDEL and DAPI immunofluorescence staining. KDEL and DAPI staining were performed using MEFs incubated at 42 °C or treated with thapsigargin (Tg) (300 nM) for 12 h. Representative images are shown. Scale bar represents 100 μm. (**d**) Cell viability was measured in MEFs treated with Tg (300 nM), Tm (1 μg/mL), or incubated at 42 °C for the indicated time periods. (**e**) Cell viability was measured in MEFs which were incubated at 42 °C for indicated time periods in the presence or absence of tauroursodeoxycholic acid (TUDCA) (2.4 mM). ** *p* < 0.01; **** *p* < 0.0001.

In order to investigate whether hyperthermic conditions induce ER stress, we examined the expression of several key UPR proteins in mouse embryonic fibroblasts (MEFs) following heat exposure for a certain time periods. Phosphorylation of eIF2α increased transiently between 1 and 2 h after heat treatment, followed by the subsequent induction of ATF4 and CHOP, as shown in Figure 1a. Tunicamycin (Tm, a *N*-glycosylation inhibitor), which is known to cause ER stress, was used as a positive control [30]. It was observed to induce eIF2α phosphorylation and expression of downstream target genes including *ATF4* and *CHOP*, as shown in Figure 1a. HSP70 expression was also induced during heat exposure, indicating that MEFs were experiencing heat stress, as shown in Figure 1a. Splicing of XBP1 was significantly enhanced and the expression of other UPR genes, *Chop* and *Grp78*, was increased during heat stress, as shown in Figure 1b. Since the major ER-resident chaperones, GRP78 and GRP94, are known to be induced by sXBP1 and ATF6α following ER stress, we examined the expression of these proteins during heat stress through immunofluorescence using an anti-KDEL antibody which detects major ER chaperone proteins including GRP78 and GRP94 [31,32]. As shown in Figure 1c, we observed that hyperthermic conditions as well as thapsigargin (Tg, SERCA inhibitor) treatment caused increased levels of those chaperones. These results indicated that hyperthermic conditions may disturb ER homeostasis and subsequently induce UPR signaling in cells.

Next, we examined whether cell death due to heat stress was related to ER stress, since ATF4 and CHOP, well-known apoptotic factors in ER stress-mediated cell death, were significantly induced by heat exposure, as shown in Figure 1a. MEFs exposed to high temperatures or treated with Tm or Tg, displayed decreased viability in a time-dependent manner, as shown in Figure 1d.

In order to assess the role of ER stress in heat stress-induced cell death, we treated MEFs with tauroursodeoxycholic acid (TUDCA), which is known as a chemical chaperone [33]. Cell viability was significantly increased at 6 and 12 h following heat exposure, in the TUDCA-treated MEFs compared to that in the control, suggesting that reduction of ER stress may protect MEFs from heat stress-induced death, as shown in Figure 1e.

### 3.2. The IRE1α Pathway Does not Protect Cells from Heat Stress-Mediated Death

**Figure 2:**
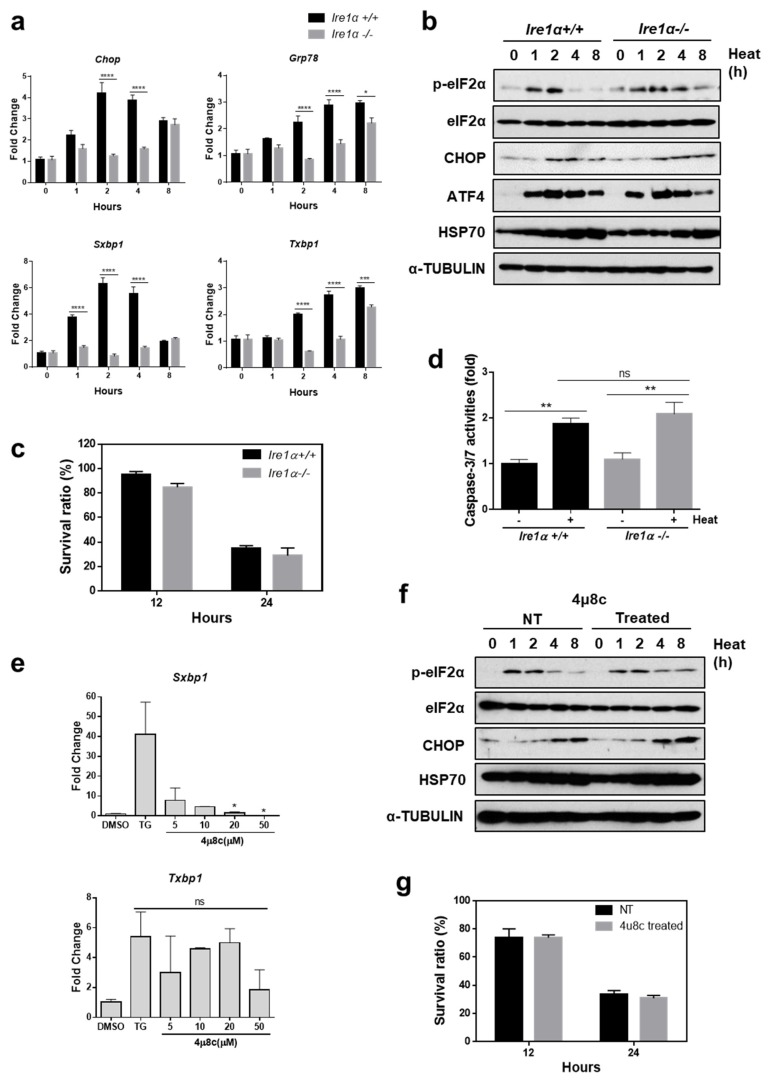
The IRE1α pathway does not protect cells from heat stress-mediated death. (**a**) Quantitative RT-PCR was performed using total RNA, extracted from *Ire1α*^−/−^ and *Ire1α*^+/+^ MEFs which were incubated at 42 °C for indicated time periods. Data are presented as means ± SEM. (n = 3 independent experiments). (**b**) Protein expression under conditions of hyperthermia was measured. Following incubation at 42 °C, cell lysates were collected at the indicated times for western blot analysis. (**c**) Cell viability was measured in *Ire1α*^−/−^ and *Ire1α*^+/+^ MEFs which were incubated at 42 °C for indicated time periods. (**d**) Caspase 3/7 activity was detected in *Ire1α*^−/−^ and *Ire1α*^+/+^ MEFs which were incubated at 42 °C for 12 h. (**e**) Expression of sXBP1 (spliced form) and tXBP (total form) were measured using MEFs treated with 4μ8c at indicated doses in the presence or absence of Tg (300 nM) for 12 h. (**f**) Protein expression under conditions of hyperthermia was measured in the presence or absence of 4μ8c (20 μM). (**g**) Cell viability was measured in *Ire1α*^−/−^ and *Ire1α*^+/+^ MEFs which were incubated at 42 °C for indicated time periods in the presence or absence of 4μ8c (20 μM). * *p* < 0.05; ** *p* < 0.01; *** *p* < 0.001; **** *p* < 0.0001.

Based on the results described above, it was assumed that ER stress may mediate heat stress-induced cell death. Since the UPR is induced to protect cells from ER stress by restoring ER homeostasis, we presumed that induction of the UPR may play a defensive role against heat stress-mediated apoptosis. Among the three branches of the UPR, we first investigated the role of the IRE1α pathway, using *Ire1α*^−/−^ and *Ire1α*^+/+^ MEFs. The levels of sXBP1 in *IRE1α*^−/−^ MEFs were markedly decreased compared to that in *Ire1α*^+/+^ MEFs, as shown in Figure 2a. In addition, we observed attenuated induction of *Grp78* and *Chop*, known to be sXBP1 target genes in *IRE1α*^−/−^ MEFs [31,34], as shown in Figure 2a. However, the pattern of phosphorylated forms of eIF2α as well as expression of CHOP and ATF4, did not show a significant difference between genotypes, as shown in Figure 2b, suggesting that the IRE1α signaling pathway was specifically disabled in *IRE1α*^−/−^ MEFs. There was no significant difference in cell viability between genotypes under hyperthermic conditions, as shown in Figure 2c. The activity of caspase 3/7 following heat stress did not show a significant difference between *Ire1α*^−/−^ and *Ire1α*^+/+^ MEFs, as shown in Figure 2d. To further confirm the role of IRE1α in heat stress-mediated cell death, we utilized 4μ8c, which selectively inhibits IRE1α RNase activity and inactivates IRE1α-mediated *Xbp1* splicing [35].

As previously reported, 4μ8c efficiently blocked splicing of XBP1 in a dose dependent manner, as shown in Figure 2e. However, there was no significant difference in induction of other branches of the UPR, as shown in Figure 2f, suggesting that 4μ8c is a specific inhibitor of the IRE1α signaling pathway. Subsequently, we checked whether inhibition of the IRE1α signaling pathway affects cell viability following heat stress. No difference in cell death was observed between MEFs treated with DMSO and 4μ8c following heat stress, as shown in Figure 2g. These results indicated that although the IRE1α pathway is induced by heat stress, it is not necessary to protect cells from heat stress-induced damage.

### 3.3. The ATF6α Pathway Does not Protect Cells from Heat Stress-Mediated Death

**Figure 3:**
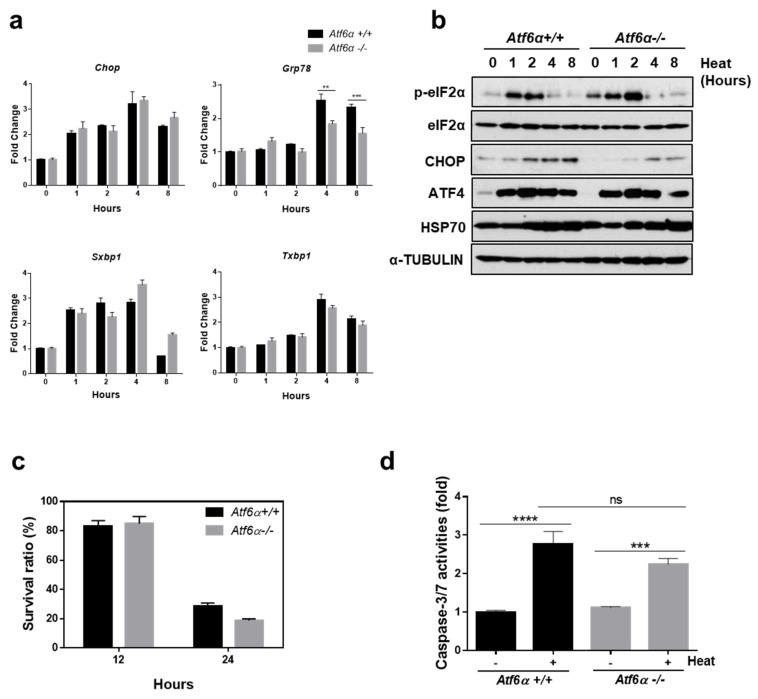
The ATF6α pathway does not protect cells from heat stress-mediated death. (**a**) Quantitative RT-PCR was performed using total RNA extracted from *Atf6α*^−/−^ and *Atf6α*^+/+^ MEFs which were incubated at 42 °C for indicated time periods. Data are presented as means ± SEM (n = 3 independent experiments). (**b**) Protein expression under conditions of hyperthermia was measured. Following 42 °C incubation, cell lysates were collected at indicated times for western blot analysis. (**c**) Cell viability was measured in *Atf6α*^−/−^ and *Atf6α*^+/+^ MEFs following incubation at 42 °C for indicated time periods. (**d**) Caspase 3/7 activity was detected in *Atf6α*^−/−^ and *Atf6α*^+/+^ MEFs which were incubated at 42 °C for 12 h. ** *p* < 0.01; *** *p* < 0.001; **** *p* < 0.0001.

Next, potential involvement of the ATF6α pathway in protecting cells from heat stress-mediated death was investigated using *Atf6α*^−/−^ and *Atf6α*^+/+^ MEFs. When cells were exposed to high temperatures, there was no significant difference between the expression of *Chop* and spliced forms of *Xbp1*, as shown in Figure 3a. However, the expression of *Grp78*, which is known to be an ATF6α target, was significantly decreased in *Atf6α*^−/−^ compared to that of *Atf6α*^+/+^, as shown in Figure 3a. Expression patterns of eIF2α phosphorylation and downstream proteins were not significantly different between genotypes, as shown in Figure 3b. There was no significant difference between the genotypes in cell viability following heat stress, as shown in Figure 3c. The activity of caspase 3/7 upon heat stress did not show a significant difference in *Atf6α*^−/−^ and *Atf6α*^+/+^ MEFs, as shown in Figure 3d. In summation, the results indicate that the ATF6α signaling pathway was not involved in protecting cells from heat stress-mediated apoptosis.

### 3.4. eIF2α Phosphorylation Is Required to Protect Cells from Heat Stress-Mediated Death

**Figure 4:**
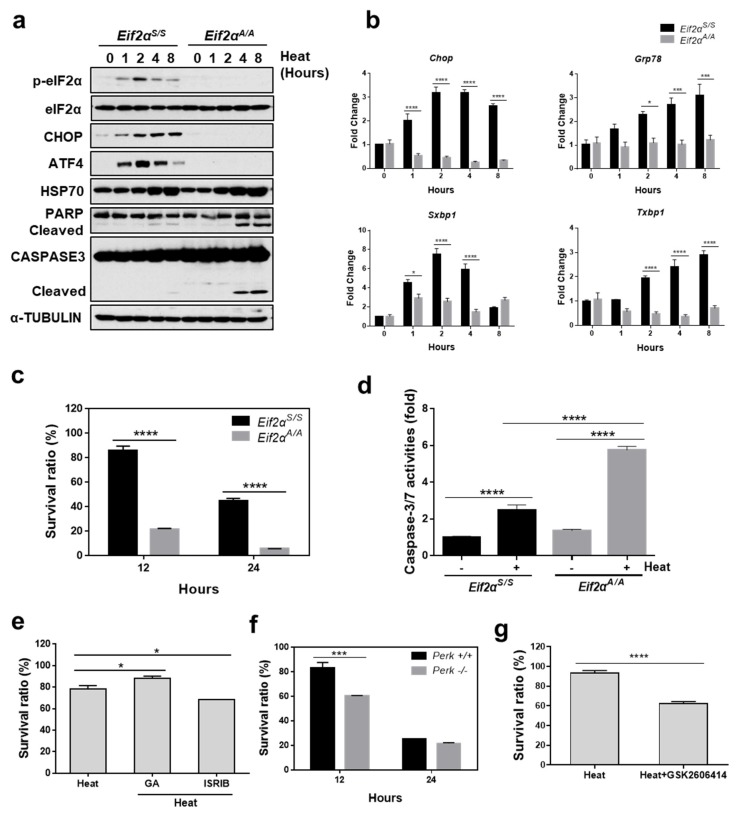
eIF2α phosphorylation is required to protect cells from heat stress-mediated death. (**a**) Protein expression was measured under conditions of hyperthermia. Cell lysates were collected from *Eif2α^S/S^* and *Eif2α^A/A^* MEFs at indicated times for western blot analysis following incubation at 42 °C. (**b**) Quantitative RT-PCR was performed using total RNA extracted from *Eif2α^S/S^* and *Eif2α^A/A^* MEFs which were incubated at 42 °C for indicated time periods. Data are presented as means ± SEM (n = 3 independent experiments). (**c**) Cell viability was measured in *Eif2α^S/S^* and *Eif2α^A/A^* MEFs which were incubated at 42 °C for indicated times. (**d**) Caspase 3/7 activity was detected in *Eif2α^S/S^* and *Eif2α^A/A^* MEFs which were incubated at 42 °C for 12 h. (**e**) Cell viability was measured in MEFs which were incubated at 42 °C for indicated time periods in the presence or absence of guanabenz (GA, 1 μM) or ISRIB (5 μM). (**f**) Cell viability was measured in *Perk^+/+^* and *Perk^−/−^* MEFs which were incubated at 42 °C for 12 and 24 h. (**g**) Cell viability was measured in MEFs which were incubated at 42 °C for 12 h in the presence or absence of GSK2606414 (100 nM). * *p* < 0.05; *** *p* < 0.001; **** *p* < 0.0001.

The role of eIF2α phosphorylation in protecting cells from heat stress-mediated cell death was investigated. For this purpose, we used a mutant MEF with a homozygous S51A mutation at the phosphorylation site in eIF2α (*Eif2α^A/A^*) and wild-type MEFs (*Eif2α^S/S^*) [36]. When MEFs were incubated at high temperatures, phosphorylation of eIF2α increased transiently between 1 and 2 h after heat exposure, followed by sequential induction of ATF4 and CHOP in *Eif2α^S/S^* but not in *Eif2α^A/A^*, as shown in Figure 4a. We also observed that the mRNA amounts of *Chop* and *Grp78* were significantly increased in *Eif2α^S/S^* but not in *Eif2α^A/A^* MEFs, as shown in Figure 4b, which is consistent with previous publications [26,37]. In addition, the spliced form of XBP1 was also highly increased in *Eif2α^S/S^* but not in *Eif2α^A/A^* MEFs, as shown in Figure 4b, suggesting that eIF2α phosphorylation and downstream signaling were completely blocked in *Eif2α^A/A^* MEFs. Next, we checked viability of *Eif2α^S/S^* and *Eif2α^A/A^* under the heat stress condition. *Eif2α^A/A^* showed ~20% viability compared to ~80% in *Eif2α^S/S^* MEFs at 12 h, which were further decreased to ~5% in *Eif2α^A/A^* and ~45% in *Eif2α^S/S^* at 24 h following heat exposure, as shown in Figure 4c. Cleavage of caspase 3 (CASP3) and PARP were clearly observed in *Eif2α^A/A^* as early as 4 h following heat exposure but not in *Eif2α^S/S^*, suggesting increased apoptosis in *Eif2α^S/S^*, as shown in Figure 4a. The enzyme activity of caspase 3/7 was also significantly increased in *Eif2α^A/A^* compared to that in *Eif2α^S/S^* at 12 h following heat exposure, as shown in Figure 4d. We further evaluated the protective effect of eIF2α phosphorylation on heat stress by modulating the eIF2α phosphorylation signaling pathway. When eIF2α phosphorylation was prolonged using guanabenz, which selectively disrupts stress-induced dephosphorylation of eIF2α [38], cell survival following heat stress was significantly improved, as shown in Figure 4e.

In contrast, when the effect of eIF2α phosphorylation was inhibited using the Integrated Stress Response Inhibitor (ISRIB) [39], cell death following heat stress was significantly increased, as shown in Figure 4e. These results strongly suggest that phosphorylation of eIF2α is required to protect cells from programmed cell death in response to heat stress.

Next, we examined whether PERK is responsible for phosphorylation of eIF2α in response to heat stress using *Perk*^+/+^ and *Perk*^−/−^ MEFs. When the cells were exposed to high temperature, *Perk*^−/−^ showed significantly reduced viability compared to *Perk*^+/+^ MEFs at 12 h, as shown in Figure 4f. To further confirm the role of PERK in heat stress-mediated cell death, we utilized GSK2606414, which selectively inhibits PERK activity and downstream signal transduction [40]. There was significantly decreased viability in MEFs when they were incubated at 42 °C for 12 h in the presence of GSK2606414 (100 nM), as shown in Figure 4g. These results suggest that PERK activation and subsequent phosphorylation of eIF2α is required for protection of cells from heat stress-mediated cell death.

### 3.5. Translational Attenuation by Eif2α Phosphorylation Is Crucial to Protect Cells from Heat Stress

**Figure 5:**
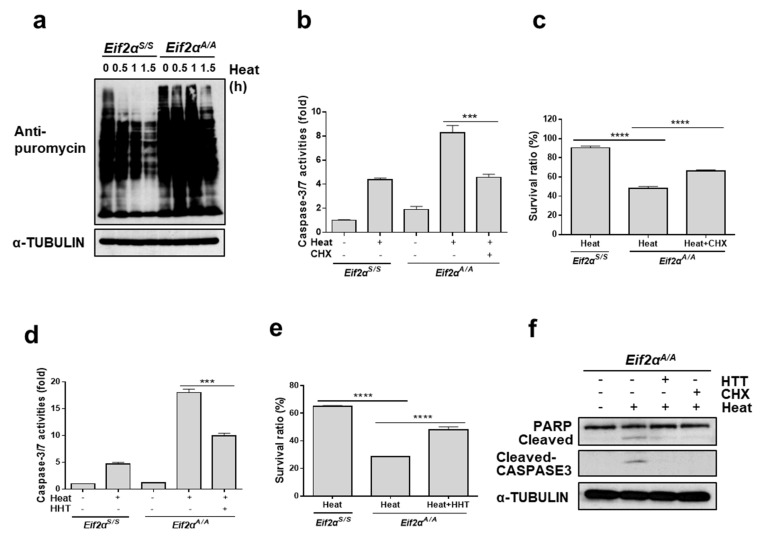
Translational attenuation by eIF2α phosphorylation is crucial to protect cells from heat stress. (**a**) Protein synthesis rate was measured using *Eif2α^S/S^* and *Eif2α^A/A^*. After incubation at indicated times at 42 °C, puromycin (10 μg) was added for 10 min, followed by western blot analysis using anti-puromycin antibody. (**b**) Caspase 3/7 activity was detected in *Eif2α^S/S^* and *Eif2α^A/A^* MEFs which were incubated at 42 °C for 12 h in the presence or absence of cycloheximide (CHX, 300 ng/mL). (**c**) Cell viability was measured in *Eif2α^S/S^* and *Eif2α^A/A^* MEFs which were incubated at 42 °C for indicated time periods in the presence or absence of cycloheximide (CHX, 300 ng/mL). (**d**) Caspase 3/7 activity was detected in *Eif2α^S/S^* and *Eif2α^A/A^* MEFs which were incubated at 42 °C for 12 h in the presence or absence of homoharringtonine (HTT, 40 nM). (**e**) Cell viability was measured in *Eif2α^S/S^* and *Eif2α^A/A^* MEFs which were incubated at 42 °C for indicated time periods in the presence or absence of homoharringtonine (HTT, 40 nM). (**f**) Cell lysates were collected from *Eif2α^A/A^* MEFs at 12 h after 42 °C incubation in the presence or absence of CHX (300 ng/mL) or HHT (40 nM). *** *p* < 0.001; **** *p* < 0.0001.

Based on the results described above, we assumed that it is eIF2α phosphorylation, and not IRE1α and ATF6α activation, which is required to protect cells from high temperature. Interestingly, there was no induction of CHOP in *Eif2α^A/A^* during heat stress whereas there was significant induction of CHOP in *Eif2α^S/S^*, as shown in Figure 4a. This result suggested that the well-known apoptotic factor CHOP is not necessary for increased cell death of *Eif2α^A/A^* following heat stress. Thus, we hypothesized that the intrinsic role of eIF2α phosphorylation, which is attenuation of protein synthesis upon stress, may be the main factor at play in this phenomenon. To assess this hypothesis, protein synthesis rates in *Eif2α^S/S^* and *Eif2α^A/A^* MEFs following heat challenge were checked using puromycin incorporation assay [41]. Translation levels were markedly decreased in *Eif2α^S/S^* but there was no significant difference from those in *Eif2α^A/A^* MEFs, as shown in Figure 5a. To assess the contribution of general repression of protein synthesis to the protection of cells from heat stress-induced apoptosis, we co-treated with cycloheximide (CHX), a potent inhibitor of translation elongation, during heat stress and checked caspase 3/7 activities. The activity was highly increased by heat stress in *Eif2α^A/A^* as shown in Figure 4d, however, it is significantly decreased when CHX was treated, as shown in Figure 5b.

The survival rate of the *Eif2α^A/A^* MEFs following heat stress was also significantly improved when treated with CHX, as shown in Figure 5c. To exclude the side effect of CHX as a chemical protein synthesis inhibitor, we employed a different protein synthesis inhibitor, homoharringtonine (HHT), which inhibits the elongation process of translation. Caspase 3/7 activity levels were markedly decreased, as shown in Figure 5d, and the survival rates were improved, as shown in Figure 5e, when *Eif2α^A/A^* MEFs were treated with HHT. In addition, cleaved forms of PARP and CASP3 were significantly decreased when treated with CHX or HTT in the presence of heat stress, as shown in Figure 5f. These results strongly indicate that translational attenuation upon heat stress is the primary defense system that protects cells from heat stress, and that eIF2α phosphorylation-mediated translational attenuation may be crucial in protecting cells from heat stress.

## 4. Discussion

In the present study, it was observed that hyperthermic conditions caused disturbance of ER homeostasis and subsequently induced the UPR. Concomitantly, cells displayed decreased viability, which was significantly improved by treatment with chemical chaperones, suggesting that ER stress caused by exposure to heat may be detrimental to cell survival. Since the UPR is a protective mechanism that restores disturbed proteostasis upon ER stress, we investigated which of the three branches of the UPR was important for protection of cells from heat stress-induced ER stress. Genetic and chemical inhibition of IRE1α signaling and ATF6α knock-out did not exacerbate cell death following heat stress. In contrast, we observed increased cell death in *Eif2α^A/A^* MEFs compared to that in wildtype *Eif2α^S/S^*. The viability of cells was significantly improved upon sustained eIF2α phosphorylation by guanabenz treatment, whereas it was significantly decreased upon inhibition of the eIF2α signaling pathway by ISRIB treatment, suggesting that eIF2α phosphorylation and downstream events are pivotal for the protection of cells from heat stress. Two well-known proapoptotic factors, ATF4 and CHOP, were unlikely to be involved in the apoptotic process following heat stress, as they were both up-regulated to similar ranges in both *Eif2α^A/A^* and *Eif2α^S/S^* MEFs. By contrast, we observed that cell viability was significantly enhanced when protein synthesis was attenuated by translation inhibitors such as CHX or HTT. These results suggest that translational attenuation by eIF2α phosphorylation following heat stress may contribute to the protection of cells from heat-mediated apoptosis.

Global translation is reduced in response to most types of unfavorable and stressful conditions. Considering that translation consumes a significant amount of cellular energy (estimated up to 50%, depending on the organisms), attenuation of protein synthesis results in a notable saving of cellular energy under conditions of stress [42,43]. In addition, reduction of translation might function to reduce the protein-folding burden of newly-synthesized proteins on disturbed proteostasis under stress conditions [44]. The translation process is divided into three steps: initiation, elongation, and termination. Although all three steps are subject to regulatory mechanisms, initiation is often regarded as the rate-limiting step and thus highly regulated by several mechanisms [44].

Phosphorylation of eIF2α is mediated by four different kinases, which integrate various intrinsic and extrinsic stress signals into a common pathway. Phosphorylated eIF2α represses general translation initiation by reducing the ability of eIF2 to combine with guanosine triphosphate and to transport the initiator Met-tRNA_i_^Met^ to ribosomes for initiation of mRNA translation [8]. Transient reduction in protein synthesis via eIF2α phosphorylation is a primary form of defense which protects cells from harmful conditions [45]. Preemptive eIF2α phosphorylation causes cells to be more resistant to ER stress or oxidative stress [45]. When keratinocytes were irradiated using UVB (Ultraviolet B), translational repression by eIF2α phosphorylation contributed to the protection of cells from UVB-induced cell death [46], which is consistent with our current findings.

Findings of the current study indicate that phosphorylation of eIF2α and concurrent repression of translation may protect against apoptosis caused by heat stress. Therefore, pharmacological agents that promote eIF2α phosphorylation or reduce ER stress may be helpful in treating heat stress-associated diseases, including heat stroke, as well as in the preservation of male germ cells [47].
